# Distinguishing Between Treatment-Resistant and Non-Treatment-Resistant Schizophrenia Using Regional Homogeneity

**DOI:** 10.3389/fpsyt.2018.00282

**Published:** 2018-08-06

**Authors:** Shuzhan Gao, Shuiping Lu, Xiaomeng Shi, Yidan Ming, Chaoyong Xiao, Jing Sun, Hui Yao, Xijia Xu

**Affiliations:** ^1^Department of Psychiatry, Affiliated Nanjing Brain Hospital, Nanjing Medical University, Nanjing, China; ^2^Department of Psychiatry, Nanjing Brain Hospital, Medical School, Nanjing University, Nanjing, China; ^3^Department of Radiology, Affiliated Nanjing Brain Hospital, Nanjing Medical University, Nanjing, China

**Keywords:** treatment-resistant schizophrenia, non-treatment-resistant schizophrenia, regional homogeneity, function magnetic resonance imaging, left postcentral gyrus, left inferior frontal gyrus, right fusiform

## Abstract

**Background:** Patients with treatment-resistant schizophrenia (TRS) and non-treatment-resistant schizophrenia (NTRS) respond to antipsychotic drugs differently. Previous studies demonstrated that patients with TRS or NTRS exhibited abnormal neural activity in different brain regions. Accordingly, in the present study, we tested the hypothesis that a regional homogeneity (ReHo) approach could be used to distinguish between patients with TRS and NTRS.

**Methods:** A total of 17 patients with TRS, 17 patients with NTRS, and 29 healthy controls (HCs) matched in sex, age, and education levels were recruited to undergo resting-state functional magnetic resonance imaging (RS-fMRI). ReHo was used to process the data. ANCOVA followed by *post-hoc t*-tests, receiver operating characteristic curves (ROC), and correlation analyses were applied for the data analysis.

**Results:** ANCOVA analysis revealed widespread differences in ReHo among the three groups in the occipital, frontal, temporal, and parietal lobes. ROC results indicated that the optimal sensitivity and specificity of the ReHo values in the left postcentral gyrus, left inferior frontal gyrus/triangular part, and right fusiform could differentiate TRS from NTRS, TRS from HCs, and NTRS from HCs were 94.12 and 82.35%, 100 and 86.21%, and 82.35 and 93.10%, respectively. No correlation was found between abnormal ReHo and clinical symptoms in patients with TRS or NTRS.

**Conclusions:** TRS and NTRS shared most brain regions with abnormal neural activity. Abnormal ReHo values in certain brain regions might be applied to differentiate TRS from NTRS, TRS from HC, and NTRS from HC with high sensitivity and specificity.

## Introduction

Schizophrenia, a group of diseases with a worldwide prevalence of approximately 0.3–0.7%, is characterized by positive symptoms, negative symptoms, and cognitive dysfunction. Etiology and treatment of schizophrenia remain a challenge for clinicians (1). Antipsychotic drugs are commonly used as treatment for schizophrenia. However, approximately 10–30% of patients with schizophrenia do not respond to antipsychotic drugs (2). Treatment-resistant schizophrenia (TRS) is characterized by the lack of response to two kinds of antipsychotics at doses ≥ 400 mg/day equivalents of chlorpromazine for a minimum period of 4–6 weeks, and with continuing moderate to severe psychopathology (especially positive symptoms) (3). TRS has been regarded as a severe and homogenous subgroup of schizophrenia that presents specific biological markers (4).

Evidence indicates that schizophrenia is a type of brain dysfunction measured by a functional connectivity (FC) method (5, 6). Moreover, FC differences exist between TRS and non-treatment-resistant schizophrenia (NTRS)/healthy controls (HCs) (7). Ganella et al. observed a decreased global efficiency but increased local efficiency in TRS compared with NTRS, mainly involving the fronto-temporal, temporo-occipital, and fronto-occipital connections (8). Dysconnectivities between cerebellar and prefrontal nodes were observed in TRS relative to NTRS (9). However, few studies have examined regional activity, which could be applied to differentiate TRS from NTRS. Furthermore, abnormal FC can be applied to distinguish TRS from NTRS/HCs with inconsistent results as regards concrete brain regions (8–12). Such inconsistency may be attributed to different sample sizes, scanners, analysis methods, and medications. However, markers to identify TRS from NTRS in advance remain lacking (13). Therefore, exploring some potential markers to identify TRS from NTRS is important. Improving understanding of the neurobiology of TRS would also be helpful in developing effective treatments for TRS.

In the present study, we examine abnormal regional activity, measured by a regional homogeneity (ReHo) method, in 17 patients with TRS, 17 patients with NTRS, and 29 HCs. Designed to explore the local consistency of brain activities, ReHo can reflect the similarity or synchronization of the time series of nearest neighboring voxels, usually 26 voxels (14). ReHo is based on the assumption that a voxel is temporally similar to those of its neighbors (15). Using this method, Wang et al. (16) revealed that dysfunction in the resting state of the brain in patients with TRS is mainly distributed in the prefrontal cortex. In this study, we hypothesized that abnormal neural activities in different brain regions of patients with TRS may constitute a potential biomarker to distinguish TRS from NRTS and/or HCs. Accordingly, ReHo was used to detect the local abnormality in the resting-state regional neural activities of patients with TRS or NTRS. Furthermore, receiver operating characteristic (ROC) curves were applied to explore the optimal sensitivity and specificity of abnormal ReHo values to differentiate TRS from NTRS or HCs.

## Methods

### Subjects

Seventeen right-handed patients with TRS were consecutively recruited from Affiliated Nanjing Brain Hospital, Nanjing Medical University from March 2013 to December 2014. Diagnosis of schizophrenia was confirmed by two experienced psychiatrists using the Structured Clinical Interview according to the DSM-IV criteria. Schizophrenia severity was assessed by using the Positive and Negative Syndrome Scale (PANSS) (PANSS total score≥60) (17). Right-handedness is determined by the Annett Hand Preference Questionnaire (18). All patients with TRS met the criteria of International Psychopharmacology Algorithm Protect (http://www.ipap.org). The exclusion criteria for this group included: (1) mood disorders according to the criteria; (2) delirium, dementia, other cognitive disorders, mental retardation, and mental disorders caused by physical illness or psychoactive substances; (3) brain trauma, epilepsy, or other known central nervous system organic diseases; (4) severe or unstable somatic diseases, such as malignant tumors, neuromuscular disorders, and autoimmune diseases.

Seventeen right-handed patients with NTRS were initially recruited from the same hospital at the same time. The diagnosis and exclusion criteria of NTRS were similar to those of TRS. Patients had a PANSS total score of < 60. The current social function of these patients was comparatively well. Patients with a reduction rate of more than 50% in PANSS total scores after 6 weeks of antipsychotic treatment were also included.

Twenty-nine right-handed HCs were recruited from the community through an advertisement. The HCs were screened using the Structured Clinical Interview for DSM-IV, non-patient edition. None of the HCs had serious medical or neuropsychiatric illnesses, and their first-degree relatives had no major psychiatric or neurological illnesses. The controls were matched with the two patient groups in relation to age, sex and years of education.

Participants were informed about the study procedures. Written informed consent was obtained from all participants and their legal guardians. The study was approved by the local Ethics Committee of the Affiliated Nanjing Brain Hospital, Nanjing Medical University (No. KY44, 2011).

### Scan acquisition

Magnetic resonance imaging (MRI) was acquired on with a 3.0T Siemens MRI scanner (Verio, Siemens Medical System) at Nanjing Brain Hospital. Birdcage head coil together with foam padding was provided to limit head movement. The scanning parameters were set as follow: repetition time (TR) = 2,000 ms; echo time (TE) = 30 ms; FOV = 220 × 220 mm; flip angle = 90; matrix size = 64 × 64; slice thickness = 4 mm; Gap = 0.6 mm; layers = 33; and time point = 240.

### ReHo data analysis

Image preprocessing was conducted by using statistical parametric mapping software (SPM8, Welcome Department of Imaging Neuroscience, London, UK). The steps included slice timing, head motion correction, and spatial normalization. Linear trend removing and band-pass (0.01–0.08 Hz) filtering were conducted with the REST (http://resting-fmri.sourceforge.net) software.

Regional homogeneity analysis was performed with an in-house REST software. Individual ReHo maps were generated by calculating the Kendall's coefficient of concordance (KCC) of the time series of a given voxel with those of its nearest neighbors (26 voxels) in a voxel-wise analysis. The formula for calculating the KCC value has been expounded in a previous study (14). To reduce the influence of individual variations in the KCC value, the ReHo maps were normalized by dividing the KCC of each voxel by the averaged KCC of the entire brain. Then the averaged ReHo maps were smoothed with a Gaussian kernel of 4 mm full-width at half-maximum.

### Statistical analysis

One-way analysis of variance (ANOVA) was used to compare the age and years of education among the three groups. Chi-square test was applied to compare sex distributions. Two-sample *t*-tests were conducted to compare the illness duration, onset, PANSS total scores, negative scores, positive scores, general scores, and chlorpromazine equivalent doses (Table S1) between the two patient groups.

Voxel-based comparisons of the whole-brain ReHo maps with ANCOVA were conducted in REST. Age and years of education were used as covariates to avoid any undetected age and education effects, although age and years of education were not significantly different across the three groups. Post-hoc *t*-tests were performed to identify variations across groups. Moreover, illness duration was also used as a covariate in the post-hoc *t*-test between the TRS and NTRS groups to minimize any potential influence of this variable. The resulting statistical map was set at *p* < 0.05 corrected via Gaussian random field theory (voxel significance: *p* < 0.001, cluster significance: *p* < 0.05).

ROC curves were utilized to prove the possibility that brain regions with abnormal ReHo can be used as potential biomarkers to differentiate between patients with TRS and NTRS or HCs. The ROC curve, created by plotting sensitivity and specificity for different cut-off points of a parameter, was a primary tool for evaluation of diagnostic tests. Each point on the ROC curve represents a sensitivity/specificity pair. ROC curves were drawn and area under the curve (AUC) was measured by the Statistical Package for Social Science version 24.0 (SPSS 24.0).

### Correlations between abnormal reho values and PANSS

Brain regions showing significant differences across groups were identified as regions of interest from which the mean ReHo values were extracted. For both patient groups, further correlation analyses were conducted group between abnormal ReHo values and PANSS total scores, negative scores, positive scores, and general scores after the normality of the data being assessed. The significance level was set at *p* < 0.05 (Bonferroni corrected).

## Results

### Characteristics of research samples

The three groups did not differ significantly as regards to age, sex, and years of education (Table 1). However, the TRS group had earlier onset and longer illness duration, higher PANSS scores, and chlorpromazine equivalent doses (Table S1) than the NTRS group.

**Table 1:** Demographic and clinical characteristics of participants.

		**TRS**	**NTRS**	**HCs**	***P values***
		**(*n* = 17)**	**(*n* = 17)**	**(*n* = 29)**	
Age (year)		31.24 ± 9.40	36.82 ± 9.12	32.73 ± 7.61	0.119
Sex(male/female)		10/7	9/8	16/13	0.378
Nation(Han/others)		17/0	17/0	29/0	–
handedness(right/left)		17/0	17/0	29/0	–
Education		12.24 ± 2.93	13.76 ± 3.58	14.28 ± 3.10	0.568
(year)					
Onset (year)		17.24 ± 2.19	29.18 ± 8.95	–	0.001
Duration(year)		14.00 ± 8.75	7.88 ± 4.72	–	< 0.001
PANSS	Total	97.76 ± 11.10	37.29 ± 6.84	–	< 0.001
	Positive	27.53 ± 5.95	9.53 ± 3.14	–	< 0.001
	Negative	21.05 ± 3.91	8.41 ± 1.91	–	< 0.001
	General	49.12 ± 5.54	19.24 ± 2.63	–	< 0.001
CED (mg/day)		696.47 ± 208.92	436.76 ± 237.85	–	0.002

*TRS: treatment-resistant schizophrenia, NTRS: non-treatment-resistant schizophrenia, HCs: healthy controls, CED: chlorpromazine equivalent dose, PANSS: Positive and Negative Syndrome Scale*.

### Group differences in ReHo

**Figure 1:**
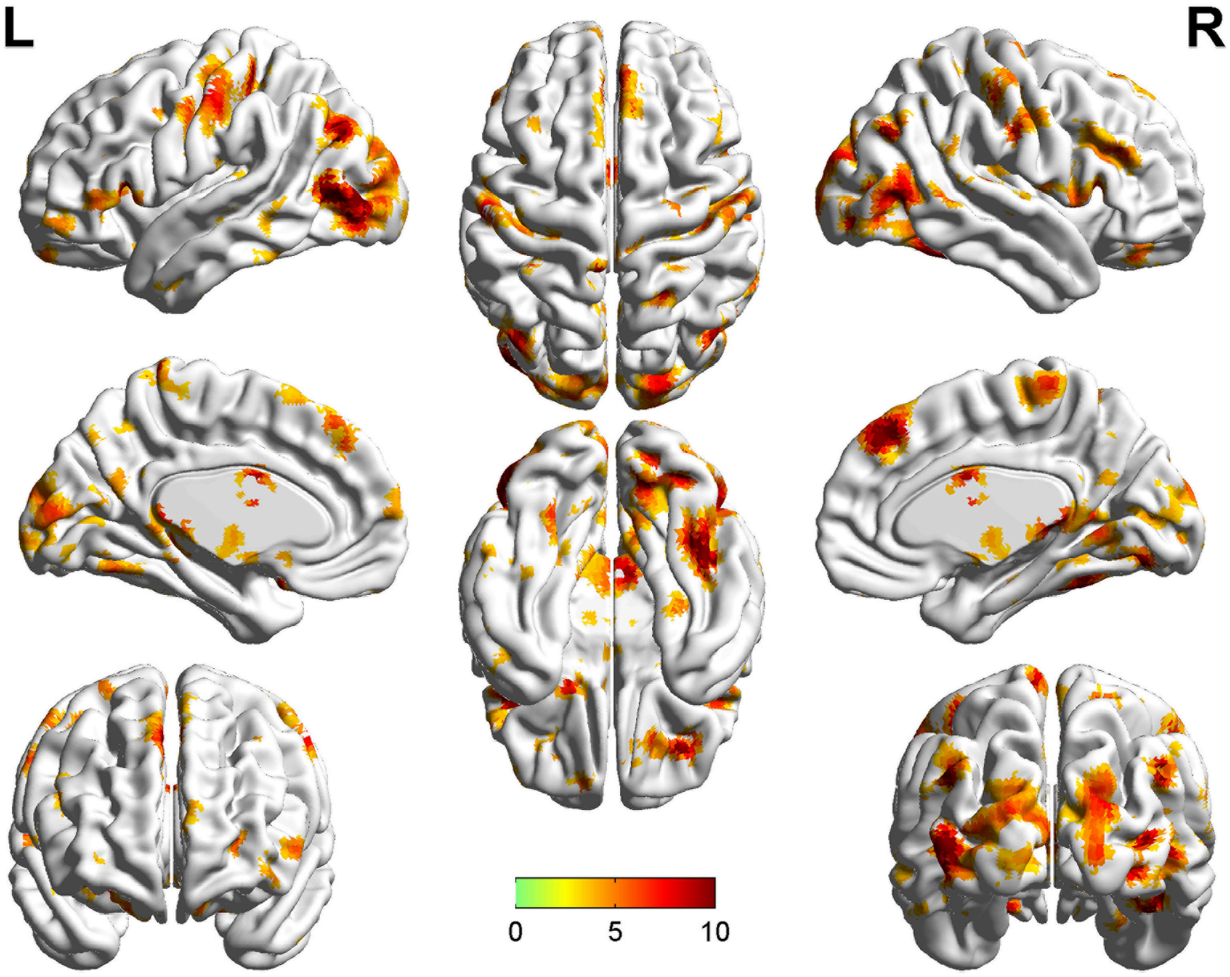
Brain regions showing significant group differences of ReHo in patients relative to controls by using ANCOVA. ReHo, regional homogeneity; ANCOVA, analyses of covariance.

**Figure 2:**
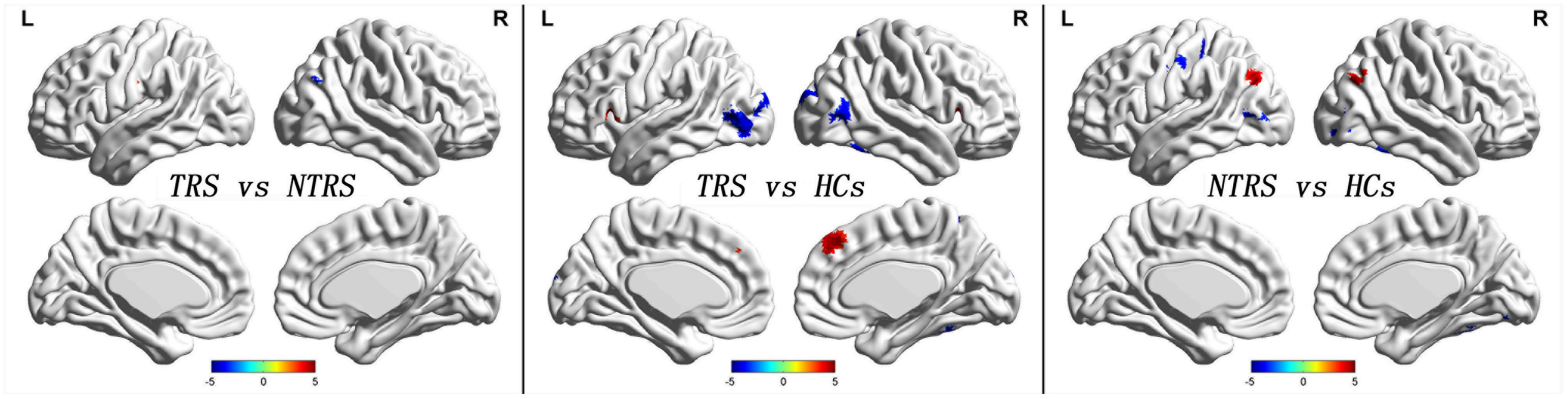
Abnormal ReHo across groups. Red and blue denote increased and decreased ReHo values. Color bar indicates *post-hoc t*-values. ReHo, regional homogeneity.

Significant group differences of ReHo in the patients relative to the controls by ANCOVA were located in the cortical and subcortical regions (Figure 1). Compared with NTRS, TRS showed increased ReHo in the left postcentral gyrus and decreased ReHo in the right angular gyrus (Table 2 and Figure 2). Compared with HCs, TRS exhibited decreased ReHo in the right fusiform gyrus, left middle occipital gyrus/middle temporal gyrus, right middle occipital gyrus/middle temporal gyrus, right superior occipital gyrus, and right superior parietal lobule, and increased ReHo in the right middle frontal gyrus/orbital part, right putamen, left inferior frontal gyrus/triangular part, right inferior frontal gyrus/triangular part, and bilateral superior medial frontal gyrus (Table 2 and Figure 2). By contrast, relative to HCs, NTRS had decreased ReHo in the right fusiform gyrus, right inferior occipital gyrus, left middle occipital gyrus/middle temporal gyrus, and left postcentral gyrus, and increased ReHo in the left angular gyrus and right angular gyrus.

**Table 2:** *Post-hoc t* tests analysis for differentiating patients from controls.

**Cluster location**	**Peak (MNI)**			**Number of voxels**	T value^*^
	**x**	**y**	**z**		
**TRS vs. NTRS**					
Left Postcentral Gyrus	−51	−12	21	42	4.4910
Right Angular Gyrus	51	−75	36	28	−4.2643
**TRS vs. HC**					
Right Fusiform Gyrus	42	−39	−18	54	−4.3036
Right Middle frontal gyrus, orbital part	30	42	−15	35	4.8364
Right Putamen	24	15	−3	47	4.4017
Left Middle Occipital Gyrus/ Middle Temporal Gyrus	−45	−72	6	224	−4.7905
Right Middle Occipital Gyrus/Middle Temporal Gyrus	48	−66	6	116	−4.3776
Left Inferior frontal gyrus, triangular part	−42	21	12	146	5.9131
Right Inferior frontal gyrus, triangular part	45	18	12	79	4.4111
Right Superior Occipital Gyrus	21	−93	21	83	−4.2365
Bilateral Superior medial frontal gyrus	3	36	45	98	5.2095
Right Superior Parietal Lobule	21	−54	63	37	−4.4150
**NTRS vs. HC**					
Right Fusiform Gyrus	42	−45	−24	69	−5.0679
Right Inferior Occipital Gyrus	33	−84	−9	147	−5.0194
Left Middle Occipital Gyrus/ Middle Temporal Gyrus	−45	−69	9	40	−3.9240
Left Angular Gyrus	−42	−72	33	80	4.7123
Right Angular Gyrus	48	−72	36	65	5.1926
Left Postcentral Gyrus	−39	−36	60	37	−4.5169

* *A positive/negative t value represents an increased/decreased ReHo; MNI = Montreal Neurological Institute; ReHo = regional homogeneity; TRS: treatment-resistant schizophrenia; NTRS: non-treatment-resistant schizophrenia; HC: healthy controls*.

### ROC analysis for differentiating patients from controls

**Figure 3:**
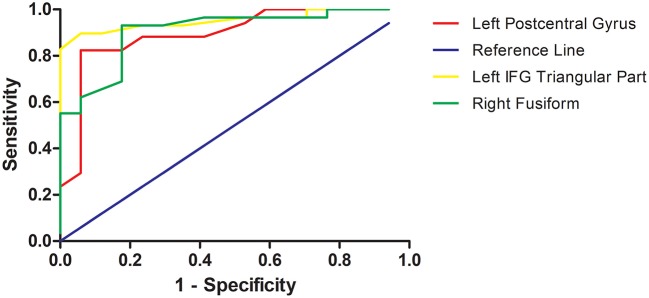
Receiver operating characteristic (ROC) curves of the optimal sensitivity and specificity by using ReHo values in the left postcentral gyrus, left inferior frontal gyrus, and right fusiform gyrus to differentiate treatment-resistant schizophrenia patients from non-resistant schizophrenia patients, treatment-resistant patients from healthy controls, and non-treatment-resistant schizophrenia patients from healthy controls, respectively. ReHo, regional homogeneity; IFG, inferior frontal gyrus.

As mentioned, several of brain regions had significant differences in ReHo across groups, thus providing a possibility that brain regions with abnormal ReHo could be used as potential biomarkers to differentiate patients with TRS from patients with NTRS or HC. To prove this possibility, the mean ReHo values were extracted from brain regions with abnormal ReHo, and ROC curves were plotted. The ReHo values in the left postcentral gyrus correctly classified 16 of 17 patients with TRS and 14 of 17 patients with NTRS, resulting in an optimal sensitivity of 94.12% and an optimal specificity of 82.35% (Table 3). Moreover, the optimal sensitivity and specificity of the ReHo values in the left inferior frontal gyrus/triangular part for differentiating TRS from HCs were 100 and 86.21%, respectively. The optimal sensitivity and specificity of the ReHo values in the right fusiform gyrus for differentiating NTRS from HCs were 82.35 and 93.10%, respectively (Figure 3).

**Table 3:** ROC analysis for differentiating patients from controls.

**Brain regions**	**Area Under the Curve**	**Cut-off point**	**Sensitivity**	**Specificity**
**DIFFERENTIATING TREATMENT-RESISTANT PATIENTS FROM NON-TREATMENT-RESISTANT PATIENTS**				
Right Angular Gyrus	0.792	−0.1746^a^	70.59% (12/17)	100% (17/17)
Left Postcentral Gyrus	0.889	−0.0966	94.12% (16/17)	82.35% (14/17)
**DIFFERENTIATING TREATMENT-RESISTANT PATIENTS FROM CONTROLS**				
Left Inferior frontal gyrus, triangular part	0.949	−0.1112	100% (17/17)	86.21% (25/29)
Right Inferior frontal gyrus, triangular part	0.856	−0.0564	70.59% (12/17)	96.55% (28/29)
Right Middle frontal gyrus, orbital part	0.872	−0.1886	76.47% (13/17)	82.76% (24/29)
Bilateral Superior medial frontal gyrus	0.844	0.1511	88.24% (15/17)	75.86% (22/29)
Right Fusiform Gyrus	0.872	−0.0830	94.12% (16/17)	75.86% (22/29)
Left Middle Occipital Gyrus/ Middle Temporal Gyrus	0.874	0.0899	100% (17/17)	68.97% (20/29)
Right Superior Occipital Gyrus	0.819	−0.0412	76.47% (13/17)	75.86% (22/29)
Right Superior Parietal Lobule	0.832	0.0334	64.71% (11/17)	93.10% (27/29)
Right Putamen	0.797	0.1480	52.94% (9/17)	96.55% (28/29)
Right Middle Occipital Gyrus/Middle Temporal Gyrus	0.888	0.0391	94.12% (16/17)	72.41% (21/29)
**DIFFERENTIATING NON-TREATMENT-RESISTANT PATIENTS FROM CONTROLS**				
Left Angular Gyrus	0.852	0.0619	94.12% (16/17)	68.97% (20/29)
Right Angular Gyrus	0.872	0.0145	94.12% (16/17)	72.41% (21/29)
Right Fusiform Gyrus	0.903	−0.1407	82.35% (14/17)	93.10% (27/29)
Right Inferior Occipital Gyrus	0.862	0.0203	70.59% (12/17)	89.66% (26/29)
Left Postcentral Gyrus	0.848	0.0176	82.35% (14/17)	79.31% (23/29)
Left Middle Occipital Gyrus/ Middle Temporal Gyrus	0.834	0.1591	94.12% (16/17)	72.41% (21/29)

a *By this cut-off point, the ReHo value in the right angular gyrus could correctly classify 12 of 17 treatment-resistant patients and 17 of 17 treatment-non-refractory patients, resulted in a sensitivity of 70.59% and a specificity of 100%. The meanings of other cut-off points were similar. ROC = receiver operating characteristic curves*.

### Correlations between ReHo values and PANSS scores in patients

The correlations between abnormal ReHo and clinical variables in the patients were examined. No correlations were observed between the ReHo values and PANSS scores in patients with TRS or NTRS.

## Discussion

In this study, the differences between TRS and NTRS occurred mainly in the parietal lobe. By contrast, the differences between TRS and HCs are widespread in the frontal, temporal, occipital and parietal lobes, while abnormal ReHo values were found in the temporal, occipital, and parietal lobes in patients with NTRS relative to HCs. Furthermore, abnormal ReHo values in the left postcentral gyrus, left inferior frontal gyrus/triangular part and right fusiform gyrus can distinguish TRS from NTRS, TRS from HC, and NTRS from HCs, respectively.

ReHo assumes that voxels within a functional brain area are more temporally homogeneous when this area is involved in a specific condition. Thus, it reflects local functional connectivity or synchronization. Increased ReHo values in patients suggest that neural function in certain regions is relatively synchronized compared with HCs (19). Nevertheless, the exact biological mechanism of an abnormal ReHo value remains unclear.

Several studies demonstrated the existence of an altered resting-state brain activity in TRS by using the ReHo method (7, 16, 20). In the present study, increased ReHo value in the left postcentral gyrus could be applied to optimally differentiate between patients with TRS and NTRS. The left postcentral gyrus belongs to the parietal lobe and the auditory and sensorimotor networks; furthermore, this area might be related to abnormal feelings (21), social cognition (22), aggression (23), and even hallucination (24, 25). Anderson et al. (26) and Quarantelli et al. (27) demonstrated that TRS had decreased GM volume in the left postcentral gyrus compared with NTRS. Furthermore, significantly decreased ReHo values in this area were observed in patients with schizophrenia (22, 28, 29), which indicated that abnormal regional activity in the left postcentral gyrus may be associated with the etiology of schizophrenia. By contrast, increased ReHo was found in the left postcentral gyrus in TRS compared with NTRS, and decreased ReHo was observed in the left postcentral gyrus in NTRS relative to HCs. In line with our findings, Gong et al. (30) revealed that Disrupted in Schizophrenia Gene 1 (DISC1) was related to the postcentral gyrus, suggesting that TRS may have distinct heredity as a special subgroup (4). Moreover, by using positron emission tomography, Monika et al. (25) discovered that the bilateral postcentral gyrus in patients with auditory hallucination showed increased metabolism, indicating that this region is a cue for explaining the increased ReHo values and symptoms.

Increased ReHo in the left inferior frontal gyrus/triangular part, which belongs to the language network and is associated with sematic processing (31), may be a potential marker to identify patients with TRS from HCs. Chyzhyk et al. and Renaud Jardri et al. (32, 33) confirmed the importance of the left inferior gyrus/triangular part for the auditory hallucination model of schizophrenia. Kubera et al. (34) detected a reduction of GM volume in the inferior frontal gyrus between schizophrenia patients who have frequent auditory verbal hallucinations and HCs. Interestingly, Jeong et al. (31) revealed a decreased activation in the inferior frontal gyrus in patients with NTRS, whereas Wolf et al. (35) and Fitzgerald et al. (36) found increased regional cerebral blood flow (rCBF) and increased brain activation in the left inferior frontal gyrus in patients with TRS. Furthermore, abnormality in this area may be associated with DISC1 in the patients, indicating enhanced heredity in TRS (30). The present findings, together with those of previous studies, imply that TRS may be regarded as a distinct subgroup of schizophrenia.

Our study revealed that decreased ReHo values in the right fusiform gyrus might be a potential biomarker for differentiating patients with NTRS from HCs. We also found decreased ReHo values in this area similar to but milder than those in TRS. The shared abnormality in the right fusiform gyrus might explain why patients with TRS and NTRS share some common symptoms. Onitsuka et al. (37) reported that the fusiform gyrus was related to object information, and faces were important and meaningful objects among visual stimuli. Moreover, Choudhary et al. (38) discovered hypoactivation and hypometabolism in the bilateral fusiform gyrus when patients with first-episode schizophrenia performed facial emotional tasks. Onitsuka et al. (37) proved that chronic schizophrenia also had hypoactivation and hypometabolism in the bilateral fusiform gyrus. A meta-analysis verified decreased ReHo in the left fusiform gyrus in patients with NTRS compared to HCs (28). In line with these studies (37, 38), the current research established that decreased ReHo values in the right fusiform gyrus can be used to distinguish patients between with NTRS from HCs.

Compared with HCs, patients with TRS showed higher ReHo values in the prefrontal cortical region, including the right middle frontal gyrus/orbital part and bilateral superior medial frontal gyrus. Decreased metabolic rate (39), decreased glutamate-glutamine to creatinine ratio (40) in the dorsolateral prefrontal cortex, and increased FC between the dorsomedial prefrontal cortex and the central opercular cortex (12) were found in patients with TRS compared with individuals with NTRS. Previously, increased ReHo values in the left medial superior frontal gyrus were negatively correlated with the patients' Characteristic of Delusion Rating Score scores but not with their delusional PANSS scores (41). These findings suggested that altered local synchronization of spontaneous brain activity may be related to the pathophysiology of delusion in schizophrenia (41).

In addition to the small sample size, our study has the following limitations. First, the effects of antipsychotic drugs should not be ignored. According to prior studies, increased regional activity and FC in the frontal gyrus, parietal lobule, temporal gyrus and striatum were observed after antipsychotic treatment (42–44). Therefore, the present results may be biased by the effects of antipsychotic drugs. Second, a longitudinal study can help us dynamically observe the changes of the ReHo values with antipsychotic drug treatment. Despite these limitations, TRS and NTRS shared the majority of brain regions with abnormal neural activity. Thus, abnormal ReHo values in certain brain regions might be applied to distinguish TRS from NTRS, TRS from HC, and NTRS from HC with optimal sensitivity and specificity.

## Author contributions

XX, SG, SL, CX, XS, YM, HY, and JS authored the manuscript. XX, SG, SL, CX, and XS collected the imaging data and clinical information. SG wrote the first draft of the manuscript. All the authors have personally reviewed the manuscript and gave final approval of the version attached.

### Conflict of interest statement

The authors declare that the research was conducted in the absence of any commercial or financial relationships that could be construed as a potential conflict of interest.
