# Supplementary material for: Distinguishing Between Treatment-Resistant and Non-Treatment-Resistant Schizophrenia Using Regional Homogeneity
Source: Front Psychiatry. 2018 Aug 6;9:282. doi: 10.3389/fpsyt.2018.00282 (PMC6088138; doi:10.3389/fpsyt.2018.00282)
Supplement: Supplementary file 1 [file Table_1.DOCX]

**Table S1** The current medication and Chlorpromazine equivalent dose of TRS or NTRS patients

|  | TRS | | |  |  | NTRS | | |
| --- | --- | --- | --- | --- | --- | --- | --- | --- |
| Subject | medication | Dose  (mg/d) | CDE  (mg/d) |  | Subject | medication | Dose  (mg/d) | CDE  (mg/d) |
| 1 | Clo | 300 | 300 |  | 1 | Zip | 100 | 375 |
| 2 | Clo+ Ris | 350+4 | 750 |  | 2 | Zip | 80 | 300 |
| 3 | Ola+Per | 15+16 | 770 |  | 3 | Ola | 10 | 300 |
| 4 | Ola+Per | 20+12 | 840 |  | 4 | Ola | 10 | 300 |
| 5 | Clo+Per | 300+20 | 700 |  | 5 | Ris | 3 | 300 |
| 6 | Ola+Hal | 10+16 | 1260 |  | 6 | Ola+Ris | 15+2 | 650 |
| 7 | Ola+Per | 15+16 | 450 |  | 7 | Ris | 1 | 100 |
| 8 | Clo+Ari | 150+20 | 550 |  | 8 | Ola | 10 | 300 |
| 9 | Clo+Ari | 250+20 | 650 |  | 9 | Ola | 10 | 300 |
| 10 | Clo+Ris | 200+4 | 600 |  | 10 | Pali | 6 | 400 |
| 11 | Clo+Per | 350+12 | 590 |  | 11 | Pali | 6 | 400 |
| 12 | Clo+Ola | 300+10 | 600 |  | 12 | Ola+Ris | 15+4 | 950 |
| 13 | Que+Per | 700+14 | 840 |  | 13 | Zip | 120 | 450 |
| 14 | Per | 36 | 720 |  | 14 | Ola+Ris | 20+4 | 1000 |
| 15 | Clo+Ris | 400+2 | 600 |  | 15 | Cendriperdone microsperes | 25mg/2 week | 400 |
| 16 | Ola+Per | 20+16 | 920 |  | 16 | Ris | 3 | 300 |
| 17 | Clo+Ola | 250+15 | 700 |  | 17 | Ola | 20 | 600 |

TRS: treatment-refractory schizophrenia, NTRS: non-treatment-refractory schizophrenia, CDE: Chlorpromazine Equivalent Dose, Per: Perphenazine, Clo: clozapine, Ris: Risperidone, Pali: Paliperidone, Ola: Olanzapine, Que: Quetiapine, Zip: Ziprasidone, Ari: Aripiprazole, Hal: Haloperidol
